# Venetoclax plus cyclophosphamide and cytarabine as induction regimen for adult acute myeloid leukemia

**DOI:** 10.3389/fonc.2023.1193874

**Published:** 2023-05-19

**Authors:** Baohang Zhang, Qingguo Liu, Junfan Li, Yimin Hu, Xin Zhao, Pingping Huang, Shangzhu Li, Ying Wang

**Affiliations:** ^1^ National Clinical Research Center for Blood Diseases, State Key Laboratory of Experimental Hematology, Haihe Laboratory of Cell Ecosystem, Institute of Hematology and Blood Diseases Hospital, Chinese Academy of Medical Sciences and Peking Union Medical College, Tianjin, China; ^2^ Tianjin Institutes of Health Science, Tianjin, China

**Keywords:** venetoclax, cyclophosphamide, acute myeloid leukemia, induction chemotherapy, cytarabine

## Abstract

**Background:**

The efficacy of induction chemotherapy (IC) for acute myeloid leukemia (AML) has improved significantly with the application of targeting drugs. Our previous study showed that a 4-day IC regimen of cyclophosphamide (CTX) and Ara-C [CA (4 + 3)] achieved similar complete remission (CR) rate (80%) compared with the traditional 7-day regimen, and the survival rate appeared to be better.

**Methods:**

In this pilot study, we further shortened the CA regimen to 3 days, added low-dose venetoclax (VEN, 200 mg/day) (VCA), and reported the efficacy and safety here.

**Results:**

Twenty-five newly diagnosed adult AML patients were enrolled in this study and evaluated for the remission rate after one cycle of the VCA regimen. The CR/Cri was 92%, and all these patients had undetectable minimal residual disease (MRD^−^). The estimated overall survival at 12 months was 79.3%. The median time for both platelet recovery and absolute neutrophil count recovery was 16 days, faster than that of traditional IC. Compared with the previous CA (4 + 3) regimen, a higher CR rate (92% vs. 80%, *P* < 0.01) and a deeper degree of remission (CR_MRD−_ rate, 92% vs. 45%, *P* < 0.01) were found in the VCA group.

**Conclusions:**

This study showed that the 3-day CTX and Ara-C regimen is highly effective in newly diagnosed AML patients, and the addition of VEN to the CA regimen achieves higher and deeper one-course remission.

## Introduction

Acute myeloid leukemia (AML) is a malignant disorder of hematopoietic stem cells. It accounts for approximately 80% of adult acute leukemia. Optimization of induction chemotherapy (IC), consolidation chemotherapy, or intensive chemotherapy to enhance the clearance of leukemia cells has the potential to improve survival. So far, anthracycline combined with cytarabine (Ara-C) is the first-line IC regimen for AML, and the complete remission (CR) rate after one or two courses of treatment is up to 80% (1, 2). Venetoclax (VEN), a selective BCL-2 inhibitor, may partly overcome the difficulty of treatment caused by the genetic heterogeneity of AML and improve the CR rate. The combination of VEN with hypomethylating agents or low-dose Ara-C in older or unfit newly diagnosed AML has shown significant improvement in overall survival (OS) (3–5). Furthermore, VEN combined with cytotoxic drugs as IC showed that CR rates could exceed 90% (6). These studies suggest that the combined chemotherapy regimens with VEN have a synergistic function.

We previously reported a 4-day IC regimen in AML that includes 4-day cyclophosphamide (CTX) and 3-day Ara-C [CA (4 + 3)] (7). The CR rate was 80%. Among the patients who completed three courses of consolidation chemotherapy, the actual 5-year disease-free survival (DFS) rate was 64%. Since the addition of VEN to IC may improve the CR rate, we further explored a 3-day CA regimen combined with a 7-day VEN (VCA) to explore whether the CR rate could be increased and preliminarily evaluated the survival rate. Correspondingly, a comparison between VCA and historical CA (4 + 3) was reported here.

## Methods

### Patients and study design

This study was carried out in the Institute of Hematology and Blood Diseases Hospital, CAMS & PUMC, between April 2021 and July 2022. Patients with newly diagnosed AML [defined by the World Health Organization (8)] were enrolled and classified into three risk groups according to the 2017 European Leukemia Net (ELN) criteria (9). The primary endpoint was CR rate, including CR and CR with incomplete blood count recovery (CRi) according to the modified International Working Group criteria (10). The secondary endpoints included overall survival (OS), minimal residual disease (MRD), response rates, event-free survival (EFS), disease-free survival (DFS), durable remissions, and adverse events. The historical CA (4 + 3) set was used for control. This study was approved by the Ethical Committee of the Institute of Hematology and Blood Diseases Hospital. Informed consent was obtained from the patients and their legal guardians in accordance with the Declaration of Helsinki.

### Treatment

A combined regimen of VEN, CTX, and Ara-C was used as induction chemotherapy. VEN was given orally at the dosage of 200 mg per day from day 1 to day 7. Ara-C (1 g/m^2^) was administered intravenously every 12 h from day 1 to day 3. CTX was administered at 20 mg/kg/day from day 1 to day 3. Posaconazole was used concomitantly to prevent invasive fungal infections and act synergistically with VEN. Patients with intermediate or poor prognosis were recommended for allogeneic hematopoietic stem cell transplantation (allo-HSCT) once the first CR was achieved. Patients unable to perform allo-HSCT were given consolidation therapy according to the NCCN guidelines. Patients remained on study for OS assessment and follow-up even if they accepted other kinds of treatment.

### Safety assessment

Adverse events (AEs) were graded according to the National Cancer Institute Common Terminology Criteria for Adverse Events version 5.0 (https://ctep.cancer.gov/protocolDevelopment/electronic_applications/ctc.htm#ctc_50). Treatment-emergent AEs, including clinical tumor lysis syndrome (TLS), were defined as those that occurred between the first dose of the study drug and 30 days after the last dose of the study drug. Clinical and laboratory TLS was defined according to the criterion reported by Howard et al. (11).

### Efficacy

Response assessment was performed between 28 and 35 days after chemotherapy, including bone marrow morphology, cytogenetics, and genetic detection. Flow cytometry was used to quantify the MRD of the marrow. Responses were defined according to the European Leukemia Net recommendations (9): CR as <5% of bone marrow blasts with normal peripheral blood counts (neutrophils ≥ 1.0 × 10^9^/L, platelets ≥ 100 × 10^9^/L) and CR with incomplete hematologic recovery (CRi, neutrophils < 1.0 × 10^9^/L, and/or platelets < 100 × 10^9^/L). Neutrophil recovery was defined as days from the start of induction therapy to neutrophil count recovered to >0.5 × 10^9^/L. Platelet recovery was defined as days from the start of chemotherapy to platelets recovered to >20 × 10^9^/L for twice evaluation without platelet transfusion.

### Statistical analysis

SPSS (version 25.0, Chicago, IL, USA) was used for the statistical analysis. Comparisons between categorical variables were performed with the *χ* ^2^ test or Fisher’s exact test. The differences between continuous variables were compared using the *t*-test or the Mann–Whitney *U* test. Overall survival was evaluated by the Kaplan–Meier method, and the statistical differences between the two groups were evaluated using the log-rank test. *P <*0.05 was defined as statistically significant.

## Results

### Patients’ characteristics

Twenty-five patients at a median age of 47.4 (range 27–68) years were enrolled in this study, consisting of 16 men (64%) and 9 women (36%). The clinical characteristics of the patients are shown in **Table 1**. All patients were diagnosed with *de novo* AML. Twenty-three patients had complications at admission. Infections were the most common, including six pulmonary infections (two hemoptysis), three invasive fungal infections, four pharyngeal or gingival infections, and three neutropenic fevers. Other comorbidities included cardiac insufficiency, vomiting, hypokalemia, and hypoproteinemia.

**Table 1:** Clinical characteristics of the patients.

Characteristic	VCA (*n* = 25)	CA (4 + 3) (*n* = 20)	*P*-value	
Age (years)			0.72
Median (range)	47.4 (27-68)	50 (24-69)	
Gender			0.94
Male (%)	16 (64)	13 (65)	
Female (%)	9 (36)	7 (35)	
AML type			
*De novo*	25 (100)	20 (100)	
Secondary	0 (0)	0 (0)	
ECOG performance status			0.14
0	5	6	
1	13	11	
2	4	3	
3	2	0	
4	1	0	
Blast percentage			0.23
Median (range)	64.4 (21-96.5)	55.7 (22.5-88.5)	
Fever/infection (%)	13 (52)	12 (60)	0.12
Classification (%)			0.18
NOS	6 (24)	7 (35)	
RUNX1-RUNX1T1	4 (16)	2 (10)	
CBFB-MYH11	1 (4)	1 (5)	
GATA2, MECOM (EVI1)	0 (0)	1 (5)	
NPM1	1 (4)	5 (25)	
CEBPA	5 (20)	1 (5)	
MLLT3-KMT2A	2 (8)	0 (0)	
KMT2A rearranged	4 (16)	2 (10)	
Myelodysplasia-related change	2 (8)	1 (5)	
Prior HMA treatment	0 (0)	0 (0)	
European Leukemia Network risk (%)			0.59
Favorable	11 (44)	9 (45)	
Intermediate	8 (32)	4 (20)	
Adverse	6 (24)	7 (35)	

**Figure 1:**
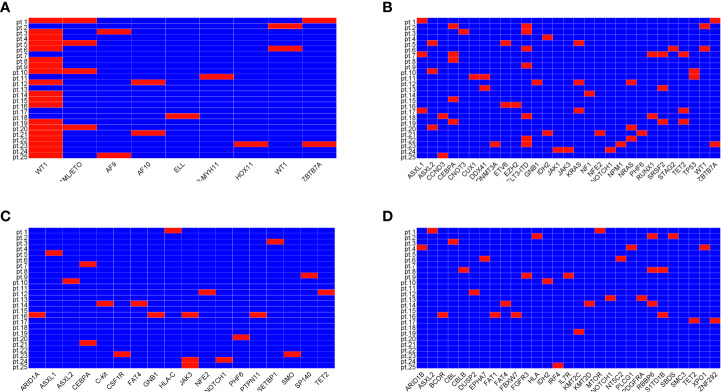
Mutations of genes related to diseases. Mutations of class II genes **(A)**, mutations of class I genes highly related to disease **(B)**, mutation of class I genes moderately related to disease **(C)**, and mutations of class I genes with unknown correlation with disease **(D)**.

The ELN risk stratification showed favorable prognosis in 44% of patients, intermediate prognosis in 32% of patients, and adverse prognosis in 24% of patients. All patients had gene mutations related to AML at diagnosis detected by next-generation sequencing (NGS) (**Figure 1**). Recurrent mutations in *RUNX1/RUNXT1*, *CBFβ-MYH11*, *NPM1*, and *CEBPA* were found in 11 patients. Three of them also carried mutations in *TP53*, *ASXL1*, and/or *RUNX1*. Another patient had mutations in *TP53*, *ASXL1*, or *KMT2A*. Mutations in the class II gene *WT1* were found in 18 patients (76%).

### Efficacy

Two patients were discharged shortly after chemotherapy due to financial reasons although their hematopoiesis had not yet recovered, and they were confirmed to have died at a later follow-up. Therefore, these two patients were evaluated as having no remission. The other 23 patients all achieved CR (including one CRi) after one course of VCA regimen and the CR rate was 92%. All those 23 patients showed MRD negativity, including the one with CRi (**Table 2**).

**Table 2:** Efficacy and adverse events between VCA and CA (4 + 3) regimen.

	VCA	CA (4 + 3)	*P*-value
Induction response (%)			<0.01
Morphologic CR	23 (92%)	16 (80%)	
MRD negative	23 (92%)	9 (45%)	
Induction failure	2 (8%)	4 (20%)	
Hematologic AEs [d, M (range)]			
The nadir of WBC (×10^9^/L)	0.02 (0.01-0.51)	0.11 (0.01-1.05)	0.002
The day of ANC recovery (> 0.5 × 10^9^/L)	16 (9-20)	17 (10-20)	0.20
The day of PLT recovery (> 20 × 10^9^/L)	16 (9-20)	16 (12-34)	0.97
Non-hematologic AEs (%)			
Angina	1 (5.3)	1 (5)	
Upper respiratory tract infection	1 (5.3)	3 (15)	
Pulmonary infections	4 (21.1)	10 (50)	
Intestinal infections	12 (63.2)	11 (55)	
Rash	0 (0)	1 (5)	
Oral ulcer	1 (5.3)	1 (5)	

WBC, white blood cell; PLT, platelet; ANC, absolute neutrophil count; AEs, adverse events.

Compared with the previous CA (4 + 3) regimen, patients who received VCA achieved a higher CR_MRD−_ rate (92% vs. 45%, *P* < 0.01). The time required for platelet recovery (≥20 × 10^9^/L) and neutrophil recovery (≥0.5 × 10^9^/L) was assessed among patients who achieved CR/CRi. The median time for both platelet recovery and neutrophil recovery was 16 (9–20) days, and it had no difference compared with that of the CA (4 + 3) group. The plasma concentration of VEN was detected in five patients and the mean level was 1,880 ng/ml.

### Survival

The last follow-up of the patients in the VCA group was in December 2022, and the median time of follow-up was 18.6 (1–20.3) months. Two patients died 1 month after IC. Three patients with adverse prognosis stratification relapsed (12%, 3/25) and finally died of the disease. Another patient died of infection during consolidation therapy. Three patients accepted allo-HSCT. By the end of follow-up, 19 patients were alive and remained CR. The median duration of OS was not reached. The estimated OS at 12 months was 79.3%.

**Figure 2:**
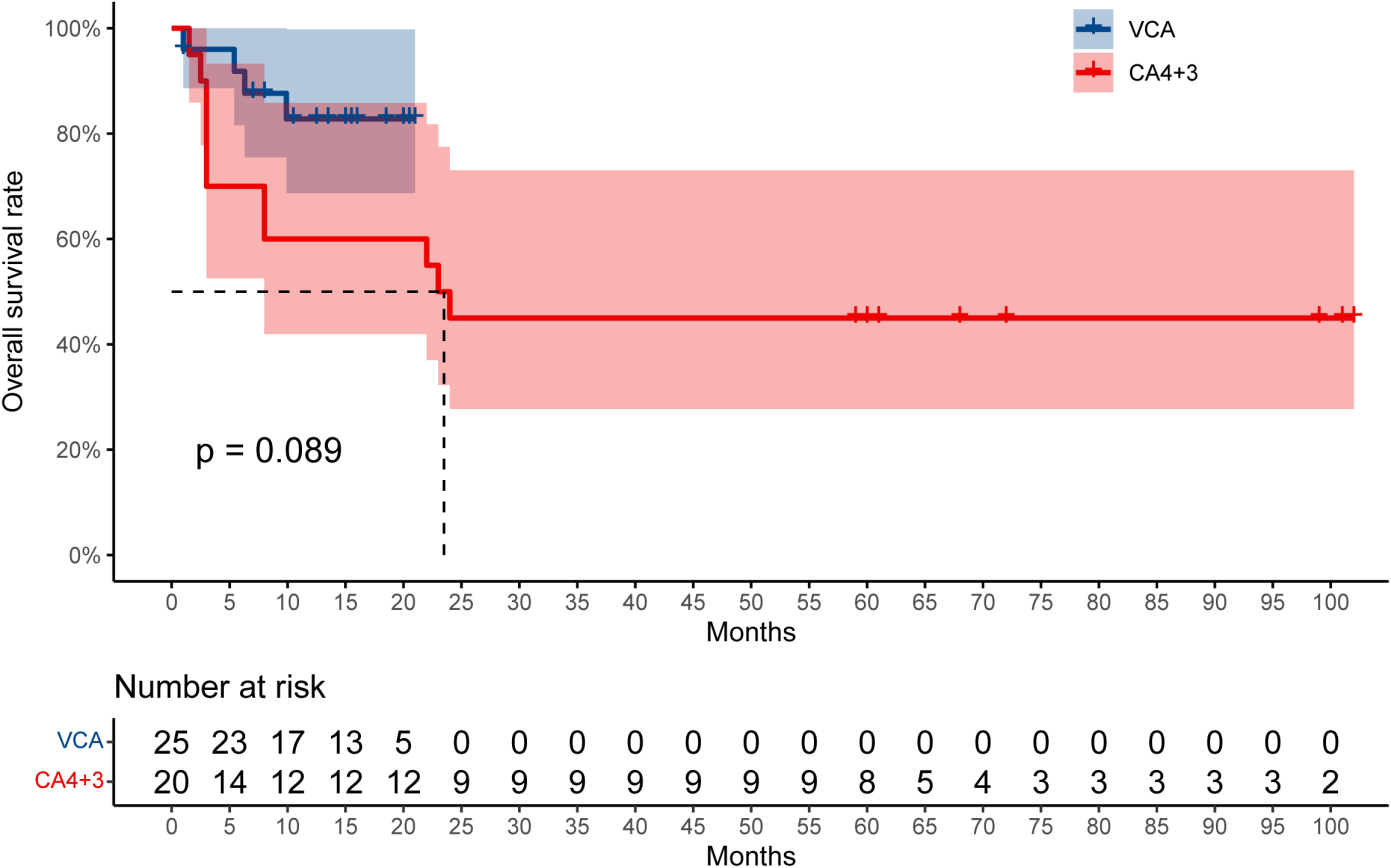
The overall survival rate between VCA and CA (4 + 3).

The overall survival rate of VCA and CA (4 + 3) is shown in **Figure 2**. We further combined the data of patients in the VCA group and the CA (4 + 3) group to analyze the predictors for OS. Multivariate analysis showed that VCA regimen use (odds ratio 0.08; 95% CI, 0.01 to 0.59, *P* = 0.013), younger age (odds ratio 1.08; 95% CI, 1.01 to 1.16, *P* = 0.025), and the ELN risk stratification of favorable prognosis (odds ratio 0.05; 95% CI, 0 to 0.6, *P* = 0.018) were associated with better survival.

### Safety

A summary of treatment-related AEs in patients who received the VCA regimen is shown in **Table 2**. Patients who received VCA had a lower count of white blood cells compared with those who received CA (4 + 3) (0.02 × 10^9^/L vs. 0.11 × 10^9^/L, *P* = 0.002). However, the infection rate and infection severity were similar between the two groups. The most common origins of infections were the intestines and lungs. Laboratory-defined TLS was found in one patient (elevations of potassium, phosphorus, and uric acid) without clinical symptoms.

## Discussion

Previous studies of VEN have shown significant improvement in OS when combined with hypomethylating agents (HMAs). A 25% reduction in the risk of death was reported with the combination of VEN, showing that VEN plus LDAC was associated with improvement in median OS (7.2 vs. 4.1 months) (12). Higher response rates and OS were reported using VEN and azacitidine (AZA) than AZA alone (response rate, 66.4% vs. 28.3%; OS, 14.7 vs. 9.6 months) (13). VEN combined with intensive chemotherapy such as CLIA and FLAG-IDA for newly diagnosed AML or high-risk myelodysplastic syndrome also showed encouraging results (3, 14, 15). In our study, the median OS was 23.5 months in the CA (4 + 3) regimen, and it was not reached in the VCA regimen (*P* = 0.089), demonstrating improved survival. The estimated OS at 12 months of the VCA regimen is 79.3%. This cohort of the study showed that the addition of VEN to the CA regimen leads to a one-course CR rate of up to 92% in adult AML, higher than that of the previous CA (4 + 3) regimen, supporting the role of VEN in improving the inducible remission rate.

A meta-analysis suggested that MRD negativity is associated with better DFS and OS in AML patients (16). Patients who achieved morphological remission but had detectable minimal diseases (MRD^+^) are at high risk of relapse (17). For patients under 40 years old, consolidation therapy containing high-dose Ara-C reduces relapse rate (18). In the current study, all the patients with CR/CRi achieved MRD negativity (100%). Compared with the one-course MRD-negative rate of 45% in the historical CA (4 + 3) group, patients who received VCA achieved deeper remission regardless of their prognostic stratification. Our results herald the ability of VEN in clearing leukemic cells (19). However, the sample size of this study is small, and further prospective study with a large sample size is required to validate the results. Nevertheless, this study showed that the combination of low-dose VEN with CA achieved deeper remission in newly diagnosed AML.

The hematologic toxicity of IC is highly associated with the duration of agranulocytosis which most likely causes life-threatening infections. The median time to ANC recovery of the VCA regimen was 16 days, much shorter than 29 days in traditional induction chemotherapy (20, 21). This result may be attributed to the short chemotherapy duration of CTX and Ara-C (3 days) and the low total dose but high blood concentration of VEN. VEN is a substrate of cytochrome P450 (CYP) 3A enzyme (CYP3A4). Posaconazole, which is used to prevent invasive fungal infections, also functions as a potent inhibitor of CYP3A4. Hence, the concomitant use of posaconazole and VEN can increase the blood concentration of VEN. The total dosage of VEN in our study is 1,400 mg, much lower than that used in previous reports (4,800, 5,600, 2,800, and 2,700 mg, respectively) (3, 6, 14, 15), but the blood concentration is high. Thus, the fast ANC recovery of VCA results in a lower percentage of severe infection and death caused by infections.

In conclusion, we demonstrate that the VCA regimen could achieve a high CR and CR_MRD−_ rate and long-term survival than traditional IC regimens in newly diagnosed AML. Patients also benefit from the shorter ANC recovery time. A prospective study with a large sample size is required to validate the results.

## Data availability statement

The original contributions presented in the study are included in the article/supplementary material. Further inquiries can be directed to the corresponding author.

## Ethics statement

This study was approved by the Ethical Committee of the Institute of Hematology and Blood Diseases Hospital. Informed consent was obtained from the patients and/or their legal guardians in accordance with the Declaration of Helsinki.

## Author contributions

QL designed the study, enrolled the patients, analyzed the data, and wrote the manuscript. BZ performed the statistical analysis and drafted the manuscript. JL, YH, PH, and SL enrolled the patients and edited the manuscript. XZ and YW analyzed the data and edited the manuscript. All authors contributed to the article and approved the submitted version.
